# Cost-effectiveness evaluation of benmelstobart, anlotinib and chemotherapy in patients with extensive-stage small-cell lung cancer

**DOI:** 10.3389/fphar.2025.1524108

**Published:** 2025-08-19

**Authors:** Jing Nie, Conghui Kou, Ke Tang, Siyu Dai, Xifeng Zhao, Jiyong Wu

**Affiliations:** ^1^ Department of Pharmacy, Shandong Second Provincial General Hospital, Jinan, Shandong, China; ^2^ College of Pharmacy, Linyi University, Linyi, Shandong, China; ^3^ College of Pharmacy, Shandong Medical College, Jinan, Shandong, China

**Keywords:** extensive-stage small-cell lung cancer, benmelstobart, anlotinib, carboplatin, etoposide, cost-effectiveness

## Abstract

**Introduction:**

Programmed death-ligand 1 (PD-L1) blockade is a growing treatment for extensive-stage small cell lung cancer (ES-SCLC). This study evaluates the cost-effectiveness of benmelstobart and anlotinib plus etoposide/carboplatin (EC) compared versus anlotinib plus EC and EC alone for patients with ES-SCLC in China.

**Methods:**

Using a Markov model over 5-year boundary and data from the ETER701 trials, we analyzed quality-adjusted life-years (QALYs), incremental cost-effectiveness ratio (ICER), total cost, incremental net health benefit (INHB) and incremental monetary benefit (INMB). To address uncertainties, we conducted one-way analysis and probabilistic sensitivity analysis (PSA). Scenario analyses were used to evaluate the resilience of our model's findings.

**Results:**

The administration of triple therapy for ES-SCLC demonstrated a significant improvement in QALY, with respective gains of 0.26, 0.39, compared with the other two schemes. However, enhanced therapeutic benefit was accompanied by increased costs. And triple therapy showed less cost-effectiveness with ICER of $189797.99 and $149249.24 per QALY respectively when compared with other schemes. Moreover, the analysis revealed an INHB of −1.04, −1.12 QALYs, and the INMB of −39755.48 $, −42819.93 $ respectively. Sensitivity analysis demonstrated that benmelstobart's cost was the main driver of cost-effectiveness. The cost-effectiveness acceptability curve displayed that the likelihood of triple therapy being cost-effective increased from 34.20% to 97.60% when the threshold value for cost per QALY gained varied from $180000 to $240000. The scenario analysis supported these findings.

**Discussion:**

Triple therapy was a less cost-effective option for patients with ES-SCLC compared with anlotinib plus EC and EC alone in China.

## 1 Introduction

Drawing from the most recent data provided by the International Agency for Research on Cancer (IARC), lung cancer has reaffirmed its status as the preeminent malignancy, with a staggering 2.481 million new cases diagnosed globally in 2022 (Bray et al., 2024). Among the various subtypes of lung cancer, small cell lung cancer (SCLC) stands out as the most invasive, constituting approximately 15% of all lung cancer cases. Its reputation is largely due to its aggressive nature and tendency for early metastasis (Rudin et al., 2021). A significant proportion, nearly two-thirds, of patients are diagnosed with extensive-stage small cell lung cancer (ES-SCLC) at the outset, facing a disheartening 5-year survival rate that hovers below 5% (Zhou et al., 2020). This grim prognosis is predominantly attributed to the high frequency of disease recurrence and metastasis (Ko et al., 2021).

Historically, platinum-based doublet chemotherapy, notably the regimen combining platinum agents with etoposide, has served as the cornerstone of first-line therapy for ES-SCLC. Yet, this strategy has only modestly extended survival, typically to about 8–10 months (Pavan et al., 2019). Recently, the therapeutic landscape has been revolutionized by the convergence of chemotherapy with immune checkpoint inhibitors, specifically those targeting the programmed death protein 1 (PD-1) and its ligand (PD-L1). A wealth of clinical research has attested to the significant survival benefits afforded by this synergistic approach. The IMpower 133 trial demonstrated that atezolizumab combined with chemotherapy achieved a median overall survival (OS) of 12.3 months in ES-SCLC patients (Horn et al., 2018; Liu et al., 2021). Similarly, durvalumab and serplulimab in combination with chemotherapy showed median OS of 12.9 months and 15.4 months, respectively, for ES-SCLC treatment (Paz-Ares et al., 2019; Goldman et al., 2021; Cheng et al., 2022). However, the specter of resistance that can emerge during treatment poses a constraint on the enduring efficacy of these interventions. T-cell exhaustion and elevated fibronectin type III domain-containing protein 4 (FNDC4) expression are independently associated with poor prognosis in lung cancer (Liu et al., 2024; Xu and Lu, 2024). Anti-angiogenic therapy, by targeting the formation of tumor vasculature, not only refines the tumor microenvironment but also potentiates the impact of immunotherapy, offering a promising strategy to counteract resistance to both chemotherapy and immunotherapy (Augustin and Koh, 2022).

Benmelstobart, a fully humanized monoclonal antibody targeting PD-L1, represents an innovative therapeutic approach developed by Zhengda Tianqing. The ETER701 trial assessed the efficacy and safety of benmelstobart in conjunction with anlotinib and standard chemotherapy for the treatment of previously untreated ES-SCLC (Cheng et al., 2024). The findings indicate that the integration of benmelstobart with anlotinib and the EC regimen significantly extended the median OS of patients to 19.3 months, compared to 11.9 months with the EC regimen. While the combination of anlotinib and EC demonstrated a trend towards survival improvement, it did not achieve statistical significance (13.3 months vs 11.9 months) (Cheng et al., 2024). Notably, the triple therapy did not exhibit a significant increase in treatment-related adverse events compared to the double therapy, suggesting that the triple therapy’s safety profile is tolerable and manageable. These insights suggest that the incorporation of anti-angiogenic therapy into immunochemotherapy may offer a potent and secure treatment strategy for ES-SCLC patients. Despite the marked therapeutic benefits of PD-L1 inhibitors and anti-angiogenic agents, their substantial costs place a considerable financial strain on patients. To date, a systematic evaluation of the economic viability of these medications is lacking. This study, therefore, seeks to conduct a comprehensive economic assessment of the triple therapy, double therapy, and chemotherapy alone from the perspective of China’s healthcare system. The goal is to inform the rational adjustment of new drug pricing and the enhancement of the medical insurance catalog, thereby alleviating the financial burden on patients, enhancing drug accessibility, and optimizing cost-effectiveness.

## 2 Methods

### 2.1 Analytical overview

The study focused its analytical lens on an envisaged cohort comprising individuals afflicted by the intricate nexus of ES-SCLC, and who had hitherto not undergone systemic therapeutic interventions. This patient profile harmonized seamlessly with the distinct demographic fabric of the ETER701 clinical trial. In the realm of this economic assessment, an intricate Markov model, demarcated into three distinct health epochs, was meticulously erected to initiate the pivotal deliberation between the therapeutic avenues paved by benmelstobart, anlotinib and chemotherapy.

**FIGURE 1:**
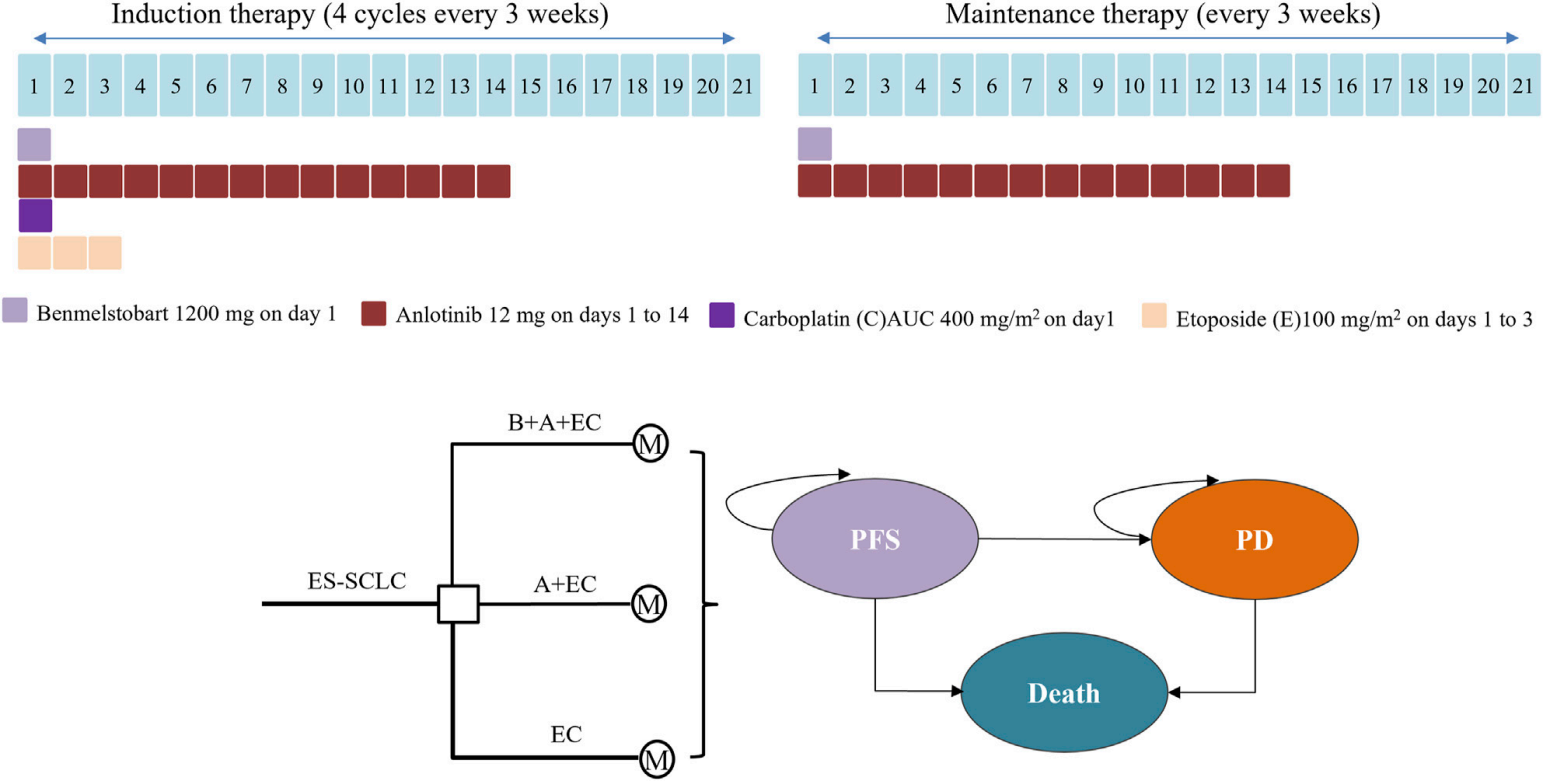
Medication regimens and model structure for ES-SCLC. ES-SCLC, extensive stage small cell lung cancer; B, benmelstobart; A, anlotinib; E, etoposide; C, carboplatin; M, Markov model; PFS, progression-free survival; PD, progressed disease.

Visualized in the illustrative construct of Figure 1, these three discrete health junctures stood as mutually exclusive entities, characterized as progression-free survival (PFS), progressed disease (PD), and the finality of mortality. The survival tenure, with its manifold implications, was meticulously parsed within these health states, further bifurcated into two avenues: one of continued vitality intertwined with PFS, and the other, an existence ensnared by the clutches of PD. The intricate calculus revealed itself as an intricate tapestry where the proportion of individuals enduring at a specific point in the cyclical timeline was adroitly deduced by the cumulative extent beneath the overarching survival curve, echoing the definitive arc of OS. Concomitantly, the contingent proportion navigating the realm of vitality entwined with PFS was elegantly ascertained through analogous calculus underpinning the PFS curve. The third facet, comprising those sustaining a tenuous coexistence with PD, was ingeniously estimated as the differential quotient between the overarching OS and the PFS trajectories.

The bedrock of this intricate proportionality found its moorings in the empirical outcomes crystallized within the annals of the ETER701 trial. This pivotal validation was realized through a harmonious juxtaposition of the model-constructed projections for PFS and OS, thoughtfully calibrated against the empirical bedrock of observed clinical data. The methodological compass of this study steadfastly adhered to the tenets prescribed by the Consolidated Health Economic Evaluation Reporting Standards (CHEERS) reporting guideline (Supplementary Table 1 in the Supplement), which orchestrate the harmonious choreography of health economic evaluations.

### 2.2 Clinical data inputs

The patient outcomes in benmelstobart, anlotinib plus etoposide/carboplatin group (B + A + EC group), anlotinib plus etoposide/carboplatin group (A + EC goup) and etoposide/carboplatin group (EC group) were influenced by findings from the ETER701 trial, at least up to the point of trial follow-up. Beyond this timeframe, standard statistical methods as outlined by Guyot et al. (2012) were employed for extrapolation. Data extraction from PFS and OS curves was conducted using GetData Graph Digitizer, version 2.20. These data points were then fitted to various parametric survival models, including Weibull, log-logistic, exponential, log-normal, Gompertz, and Generalized gamma models. Selection of the appropriate survival model was based on the Akaike information criterion (AIC) and Bayesian information criterion (BIC), with detailed goodness-of-fit outcomes presented in Supplementary Table 2 and Supplementary Table 3 within the Supplement. Our analysis concluded that the log-normal distribution provided the most accurate fit for the PFS data within the B + A + EC group. Conversely, the log-logistic distribution proved suitable for extrapolation purposes in other groups. Consequently, the combination of log-normal and log-logistic distributions was employed for the calculation of transition probabilities. To generate the parametric survival curves, the RStudio 2022.02.0 software was utilized, and the construction of the Markov model was executed using TreeAge Pro 2022. For estimation of the transition probability from PFS to mortality, we incorporated mortality rates from the general population of 2023, sourced from the mortality tables provided by the National Bureau of Statistics (Statistics, 2023).

### 2.3 Cost and utility inputs

Only direct medical expenses were taken into account, encompassing expenditures related to drug procurement, follow-up examinations, management of serious adverse effects (SAEs), and best supportive care (BSC). These details are outlined in Table 1. The monetary figures are presented in 2023 United States dollars (converted at a rate of 7.0467 RMB/USD) and were adjusted to 2023 values using the consumer price index (CPI). As indicated by the ETER701 trial report, the dosing regimen of patients receiving triple therapy (benmelstobart, anlotinib plus etoposide/carboplatin), dual therapy or chemotherapy alone were shown in Figure 1. Treatment cycles persisted for 21 days each, until factors such as unacceptable toxicity, consent withdrawal, disease progression, or investigator judgment necessitated a halt. Prices for benmelstobart, anlotinib, carboplatin, and etoposide were sourced from Shandong drug and medical consumables procurement management subsystem (https://ypjc.ybj.shandong.gov.cn/). Local medical institution rates were employed for follow-up expenses, while additional cost data were referenced from relevant literature. Discontinuations arising from SAEs were not factored into this assessment. The analysis encompassed expenses linked to managing grade 3 or higher adverse events (AEs), extracted from pertinent sources (see Table 1).

**TABLE 1:** Model parameters: baseline values, ranges, and distributions for sensitivity analysis.

Parameter	Expected value	Range	Distribution	Source
Drug costs ($)				
Anlotinib/cycle	563.24	450.59–675.89	gamma	Local charge
Carboplatin injection/cycle	74.50	59.60–89.40	gamma	Local charge
Etoposide injection/cycle	134.53	107.62–161.44	gamma	Local charge
Benmelstobart	3,485.32	2,788.26–4,182.38	gamma	Local charge
AEs costs ($)				
Neutropenia	16,619.16	13295.33-19942.99	gamma	Ding et al. (2021)
Leukopenia	16,619.16	13295.33-19942.99	gamma	Ding et al. (2021)
Thrombocytopenia	2,674.01	2139.20-3208.81	gamma	Ding et al. (2021)
Anemia	510.35	408.28–612.42	gamma	Xiang et al. (2023)
Hypertension	14.45	11.56–17.34	gamma	Dai et al. (2024)
Follow up monitoring cost ($)				
Contrast CT	447.02	357.61–536.42	gamma	Local charge
Cranial MRI	248.34	198.67–298.01	gamma	Local charge
Cervical lymph node Ultrasound	19.87	15.89–23.84	gamma	Local charge
Tumor marker	72.52	58.01–87.02	gamma	Local charge
Bone scan	120.62	96.50–144.75	gamma	Local charge
Complete blood count	2.70	2.16–3.24	gamma	Local charge
Blood-biochemistry	30.09	24.07–36.10	gamma	Local charge
Best supportive care	1,543.46	1234.77-1852.15	gamma	Zhu et al. (2023)
Utility				
PFS	0.84	0.67–0.88	beta	Ding et al. (2021)
PD	0.47	0.38–0.57	beta	Ding et al. (2021), Xiang et al. (2023)
Disutility due to AEs				
Neutropenia	0.09	0.07–0.11	beta	Ding et al. (2021)
Leukopenia	0.09	0.07–0.11	beta	Ding et al. (2021)
Thrombocytopenia	0.20	0.16–0.24	beta	Zhu et al. (2022)
Anemia	0.07	0.06–0.09	beta	Xiang et al. (2023)
Hypertension	0.04	0.03–0.05	beta	Dai et al. (2024)
Probabilities, %				
Benmelstobart + anlotinib + EC				
Neutropenia	0.70	0.63–0.76	beta	Cheng et al. (2024)
Leukopenia	0.38	0.34–0.42	beta	Cheng et al. (2024)
Thrombocytopenia	0.50	0.45–0.55	beta	Cheng et al. (2024)
Anemia	0.24	0.22–0.26	beta	Cheng et al. (2024)
Hypertension	0.16	0.14–0.17	beta	Cheng et al. (2024)
Anlotinib + EC				
Neutropenia	0.73	0.66–0.80	beta	Cheng et al. (2024)
Leukopenia	0.31	0.28–0.34	beta	Cheng et al. (2024)
Thrombocytopenia	0.54	0.48–0.59	beta	Cheng et al. (2024)
Anemia	0.27	0.24–0.29	beta	Cheng et al. (2024)
Hypertension	0.12	0.11–0.13	beta	Cheng et al. (2024)
EC alone				
Neutropenia	0.69	0.62–0.76	beta	Cheng et al. (2024)
Leukopenia	0.35	0.31–0.38	beta	Cheng et al. (2024)
Thrombocytopenia	0.36	0.32–0.39	beta	Cheng et al. (2024)
Anemia	0.24	0.21–0.26	beta	Cheng et al. (2024)
Hypertension	0.02	0.01–0.02	beta	Cheng et al. (2024)
Discount	0.05	0.00–0.08	beta	China Guidelines for Pharmacoeconomic Evaluations. (2020)

For each health state, a health utility preference was assigned on a scale ranging from 0 (indicating death) to 1 (representing perfect health). The utility values for the PFS and PD states concerning ES-SCLC were determined as 0.84 and 0.47 (Ding et al., 2021; Xiang et al., 2023), respectively. The analysis took into account the disutility values caused by severe side effects (grade 3/4 adverse effects). The assumption was made that all AEs occurred during the initial treatment cycle. Owing to the low 5-year survival rate, our analysis was conducted over a 5-year timeframe, encompassing about 90 cycles.

### 2.4 Base-case analysis

The incremental cost-effectiveness ratio (ICER) was computed as the added cost per extra quality adjusted life-year (QALY) gained, comparing each group pairwise. If the ICER fell below the predetermined threshold for acceptable expenditure ($38070.59 per additional QALY achieved), it was considered cost-effective in line with recommendations. Costs and QALYs were discounted at an annual rate of 5% to account for future values. Additionally, we derived the incremental net health benefit (INHB) and incremental monetary benefit (INMB) using the following expressions: INHB(λ) = (μ_E1_ − μ_E0_) − (μ_C1_ − μ_C0_)/λ = ΔE − ΔC/λ and INMB(λ) = (μ_E1_ − μ_E0_) × λ − (μ_C1_ − μ_C0_) = ΔE × λ−ΔC, where μ_Ci_ and μ_Ei_ represented the cost and effectiveness of each group i represents evaluation group, and 0 represents control group). The parameter λ represented the willingness-to-pay (WTP) threshold (Craig and Black, 2001).

### 2.5 Sensitivity analysis

To thoroughly validate the integrity of our foundational findings, we undertook comprehensive sensitivity analyses encompassing both one-way sensitivity assessments and probabilistic sensitivity analysis (PSA). One-way sensitivity analysis is an analytical method that evaluates the impact of individual model parameters on study outcomes by systematically varying each parameter within a predefined range. The one-way sensitivity analyses were executed across all parameters, with the parameter range derived from either reported or estimated 95% confidence intervals from the referenced studies, or determined by a considered 10% or 20% deviation from the base-case value (outlined in Table 1). Probabilistic sensitivity analysis was conducted through 1,000 Monte Carlo simulations, where all model parameters were randomly sampled within predefined distributions. The outcome measures from these multiple simulations were analyzed to evaluate the robustness of the model. The choice of distribution was guided by a gamma distribution for cost parameters and a beta distribution for probability, proportion, and preference value parameters. Drawing insights from the data amassed through these 1,000 iterations, a cost-effectiveness acceptability curve emerged, presenting the probability that triple therapy would qualify as cost-effective across various thresholds of WTP concerning health advancements (QALYs).

### 2.6 Scenario analysis

We meticulously evaluated the resilience of our model’s findings through a series of scenario analyses. Initially, we explored the potential impact of China’s pharmaceutical policies on the pricing of benmelstobart, hypothesizing a significant reduction in drug costs by either 50% or 90%. Subsequently, we delved into the ramifications of discounts, ranging from 3% to 8%, on pharmacoeconomic outcomes, aligning with the parameters stipulated in the China Guidelines for Pharmacoeconomic Evaluations. (2020). Our comprehensive data analysis was expertly executed utilizing TreeAge Pro 2022, ensuring the accuracy and reliability of our insights.

## 3 Results

### 3.1 Base-case analysis

The 21-day therapy costs were as follows: anlotinib ($563.24), carboplatin ($74.50), etoposide ($134.53) and benmelstobart ($3,485.32) (Table 1). In comparison with chemotherapy therapy, both triple therapy and dual therapy showed less cost-effectiveness, with ICER of $149225.65 and $62961.20 respectively. While compared with dual therapy, triple therapy also showed less cost-effectiveness, with ICER $189763.99. The findings for both INHB and INMB showed negative values, suggesting that neither the triple therapy nor the dual therapy offered a higher cost-effectiveness ratio (Table 2).

**TABLE 2:** Base results of triple therapy, dual therapy or chemotherapy alone.

Strategy	Cost	Incr cost	Eff	Incr eff	ICER	INHB, QALY	INMB, $	Remarks
EC	46,771.61	NA	0.45	NA	NA	NA	NA	NA
A + EC	54,523.56	7,751.95	0.57	0.12	62,959.34	−0.08	−3,064.46	compared with EC
B + A + EC	104,254.26	57,482.64	0.83	0.39	149,249.24	−1.12	−42819.93	compared with EC
B + A + EC	104,254.26	49,730.70	0.83	0.26	189,797.99	−1.04	−39755.48	compared with A + EC

Abbreviations: E, etoposide/carboplatin; A, anlotinib; B, benmelstobart; INHB, incremental net health benefit; INMB, incremental net monetary benefit; NA, not applicable; QALY, quality-adjusted life-years.

### 3.2 Sensitivity analysis

**FIGURE 2:**
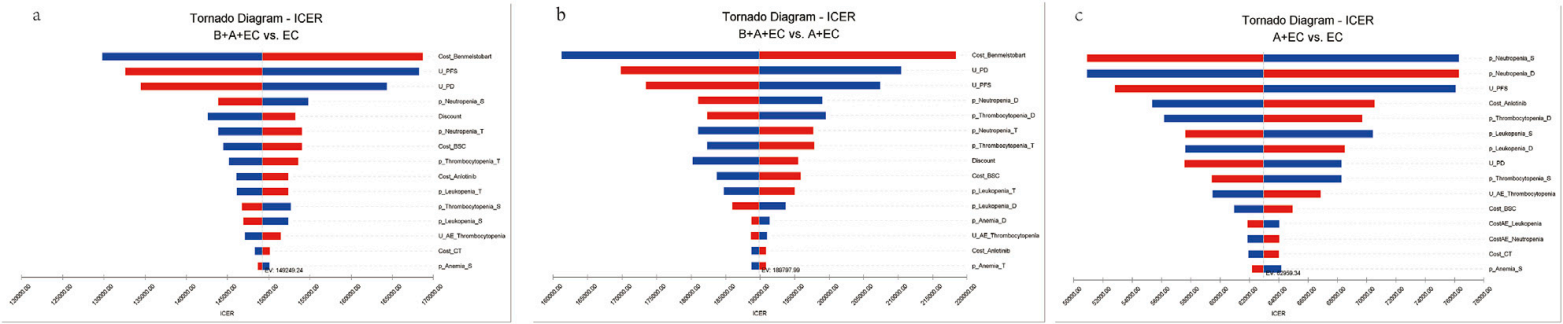
Tornado diagram of triple therapy, dual therapy or chemotherapy alone in the one-way deterministic sensitivity analysis. **(a)** B + A + EC vs. EC; **(b)** B + A + EC vs. A + EC; **(c)** A + EC vs. EC. B, benmelstobart; A, anlotinib; E, etoposide; C, carboplatin; U, utility; PFS, progression-free survival; PD, progressed disease; BSC, best supportive care; AE, adverse effect; p, probability; S, single therapy; D, dual therapy; T, triple therapy.

The one-way sensitivity analyses demonstrated that the cost of benmelstobart was a pivotal determinant of the model’s outcomes for the triple therapy group, exerting a more pronounced influence compared to the other treatment groups (Figure 2). The utility of PFS and PD were also important determinant among 3 groups. Additionally, the substantial impact of adverse reactions (AEs) in this study can likely be attributed to the elevated likelihood of severe grade 3 and higher AEs, with certain occurrences, such as Neutropenia, exceeding a staggering 70%. Adopting a lower boundary value for the cost of benmelstobart (i.e., $2,788.26) resulted in an ICER of $129786.80 per additional QALY gained for triple therapy compared with chemotherapy. Conversely, adopting an upper boundary ($4,182.38) yielded an ICER was $168711.68 per additional QALY gained. However, none of these changes surpassed the threshold of $38070.59/QALY, which was the predetermined limit. Other parameters, including probability of AEs, discount, and the cost of best support care, AEs or laboratory tests, exhibited only moderate or weak associations with the outcome and did not lead to ICERs exceeding the threshold.

**FIGURE 3:**
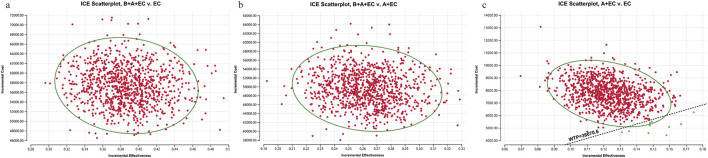
1,000 Monte Carlo simulation diagram of the probabilistic sensitivity analysis. ICE, ICE, incremental cost-effectiveness. **(a)** B + A + EC vs. EC; **(b)** B + A + EC vs. A + EC; **(c)** A + EC vs. EC. B, benmelstobart; A, anlotinib; E, etoposide; C, carboplatin.

**FIGURE 4:**
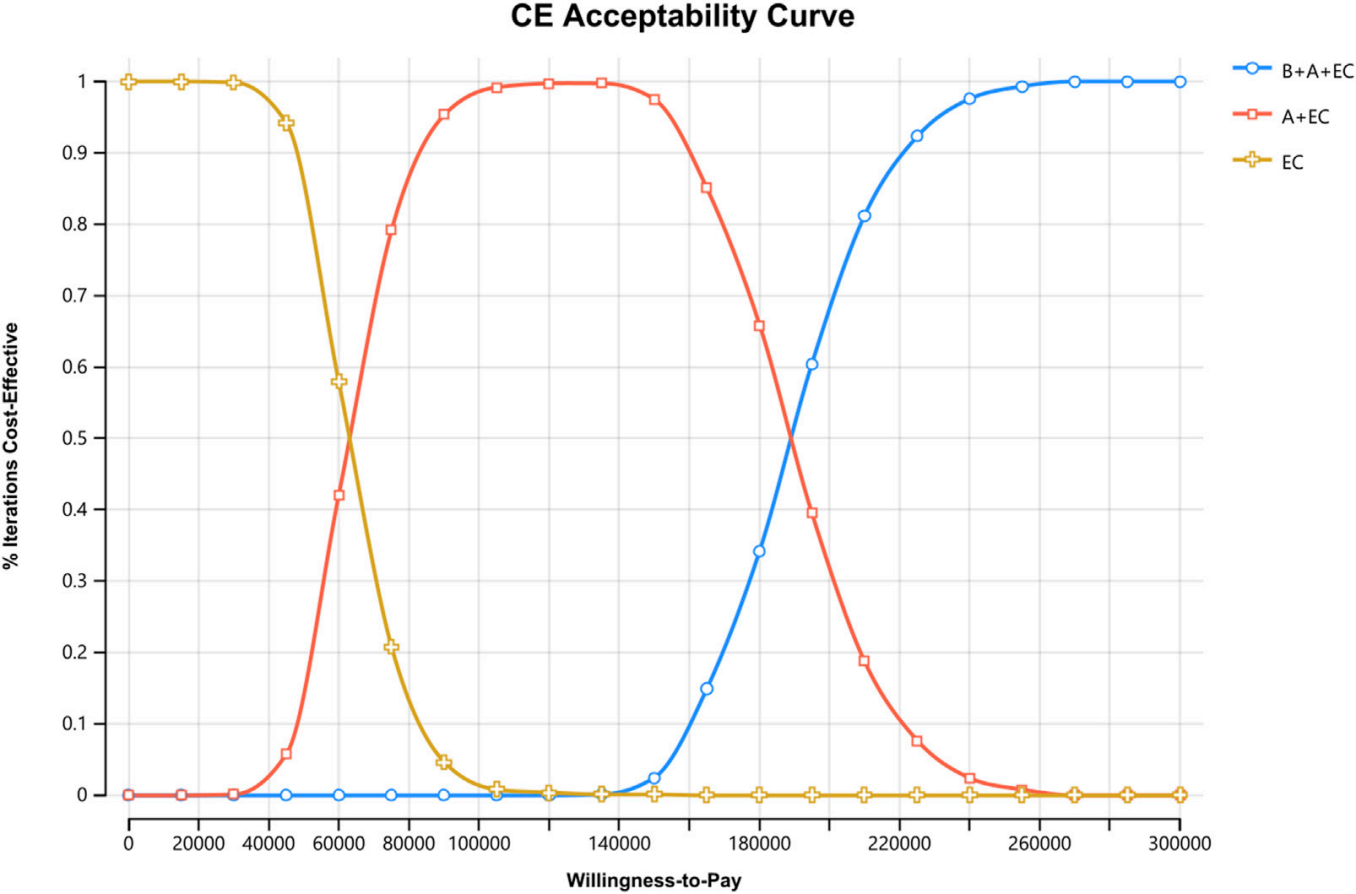
Cost-effectiveness Acceptability Curves for Patients with ES-SCLC. CE, cost-effectiveness, B, benmelstobart; A, anlotinib; E, etoposide; C, carboplatin.

Compared to EC chemotherapy, the probabilistic sensitivity analysis revealed that the combination of benmelstobart and anlotinib contributed a mean increase of 0.39 QALYs (ranging from 0.30 to 0.49) and an additional average cost of $57482.64 (with a range of $46541.86 to $71511.23). Consequently, the calculated mean ICER was $149249.24/QALY (from $145941.29/QALY to$155139.53/QALY). As demonstrated in Figure 3, each group exhibited a negligible or minimal likelihood of achieving cost-effectiveness. Moreover, the cost-effectiveness acceptability curve displayed that the likelihood of triple therapy being cost-effective increased from 34.20% to 97.60% when the threshold value for cost per QALY gained varied from $180000 to $240000 (Figure 4).

### 3.3 Scenario analysis

The scenario analysis outcomes are presented in Table 3. Notably, a 90% reduction in the price of benmelstobart results in an ICER of $61667.75 compared with EC, which remains above the WTP threshold, set at three times the GDP *per capita*, suggesting potential decreased cost-effective. Additionally, when the discount rates are adjusted to 3% and 8%, the ICER for benmelstobart in comparison to chemotherapy is calculated to be $146580.26 and $153263.18, respectively. These results clearly demonstrate that benmelstobart is less cost-effective across both discount rates. It is important to highlight that the impact of discounts on the outcomes is relatively minor, and only substantial decreases in drug’s prices could potentially make benmelstobart a more economically viable option.

**TABLE 3:** Scenario analysis results.

No.	Strategy	Cost	Incr cost	Eff	Incr eff	ICER	Remarks
1	EC	46,771.61		0.45			price dropped by 50%
	B + A + EC	85,514.48	38,742.87	0.83	0.39	100,592.85	
2	EC	46,771.61		0.45			price dropped by 90%
	B + A + EC	70,522.66	23,751.04	0.83	0.39	61,667.75	
3	EC	45,991.93		0.44			8% discount
	B + A + EC	101,796.73	55,804.81	0.80	0.36	153,263.18	
4	EC	47,326.70		0.46			3% discount
	B + A + EC	106,020.64	58,693.94	0.86	0.40	146,580.26	

## 4 Discussion

With the rising incidence of ES-SCLC nationwide, the associated costs are also progressively on the rise. Multiple studies have examined the cost-effectiveness of novel anti-cancer drugs for ES-SCLC treatment. Comparatively, the economic findings for durvalumab in combination with platinum/etoposide and chemotherapy alone suggest them to be less cost-effective regimen from the United States healthcare system perspective (Ding et al., 2021). In Xiang’s study, serplulimab plus chemotherapy was determined to be lacking in cost-effectiveness, and it was suggested that cost-effectiveness may improve with price discounts on serplulimab from the perspective of Chinese healthcare system (Xiang et al., 2023). Adebrelimab plus chemotherapy was not an economical strategy compared with chemotherapy for first-line treatment of ES-SCLC in China, however, You’s study held a different view (You et al., 2022; Long et al., 2024).

The ICER represents the additional cost per QALY gained when comparing two treatment strategies. When the ICER exceeds the cost-effectiveness threshold, the intervention is typically deemed not cost-effective due to the excessive financial burden on patients or healthcare systems relative to the clinical benefit achieved. Our study addresses the unmet need for an economic assessment of benmelstobart triple therapy. Drawing on data from the ETER701 trial, our analysis demonstrates the less cost-effectiveness of triple therapy for the treatment of advanced ES-SCLC at a WTP threshold of $38070.59 per QALY. These findings are generally robust and supported by the results of both one-way sensitivity and probabilistic sensitivity analysis. The results of scenario analysis show that adjusting the discount rate or lowering the price of benmelstobart still support the outcomes of base-case analysis. This may be due to the high cost of benmelstobart, which has a crucial influence on the model results.

The prevention of disease progression by the combination of benmelstobart triple therapy played a crucial role in determining the economic outcomes. The results from the 1-way sensitivity analysis demonstrated that the cost of benmelstobart was the most influential factor. Additionally, the utility of PD, and utility of PFS were also deemed significant. Furthermore, the PSA results indicated that the ICER values of triple therapy were predominantly situated above the threshold curve when compared with the other treatment groups, which was set at 3 times China’s *per capita* GDP. Benmelstobart triple therapy is also found to not be a cost-effective option.

To the best of our knowledge, this study represents the inaugural analysis to comprehensively examine the economic implications of administering benmelstobart triple therapy for the management of advanced ES-SCLC. By leveraging an economic modeling methodology, we have synthesized the most up-to-date evidence available. Despite this milestone, we must acknowledge the lack of sufficient data pertaining to the economic outcomes associated with immune checkpoint inhibitors (ICIs) for the treatment of advanced ES-SCLC. Further investigation is warranted to ascertain the specific patient cohorts that would drive optimal benefits from the administration oftriple therapy.

The analysis at hand does exhibit certain limitations that warrant consideration. Firstly, due to the absence of head-to-head data, we were unable to incorporate other ICIs like atezolizumab, durvalumab as first-line treatments, despite their demonstrated positive health outcomes for patients with advanced ES-SCLC. Thus, it is essential that our analysis be updated once first-line data become available. Secondly, in this study, we employed parameter distribution fitting to extrapolate the PFS and OS curves. Although this allows for the extrapolation of survival trends beyond the ETER701 study period, it also introduces uncertainty into the model results. Therefore, our next step should involve further exploration of the clinical efficacy of triple therapy through real-world research, as well as validation of the economic feasibility of this regimen with the aid of more mature survival curves from longer follow-up results in the ETER701 study. Thirdly, it is important to acknowledge that in real-world scenarios, individual patient characteristics may lead to variations in subsequent treatment plans. While this study was based on data from the ETER701 clinical trial, the model may not fully capture the heterogeneity of individual patient characteristics. Although sensitivity analyses were performed across predefined parameter ranges to address uncertainty, the complexity of real-word patient profiles remains incompletely represented. Future research should focus on developing more sophisticated models that integrate individualized patient characteristics, comorbidities, and relevant clinical indicators. Such enhanced modeling approaches would enable the generation of personalized, economically optimized treatment recommendations that better reflect clinical decision-making processes in routine practice. Fourthly, due to the lack of time series data, the present analysis did not account for variations in costs associated with survival time and duration, such as follow-up costs. Lastly, in clinical practice, grade 1–2 AEs typically require minimal or no intervention and incur relatively low management costs. However, their exclusion from economic evaluations may lead to underestimation of total treatment costs and potential overestimation of a regimen’s cost-effectiveness. Nevertheless, this limitation is unlikely to significantly alter the study conclusions. In real-world clinical practice, it is essential to adopt a patient-centered approach that integrates individual patient characteristics, AE management costs, and treatment cost-effectiveness to optimize therapeutic option for each patient. Moreover, since this evaluation reflects the general clinical practice for managing advanced ES-SCLC, it can serve as a valuable reference for physicians and policymakers. Furthermore, pharmacoeconomic analyses provide substantial, objective, and comprehensive evidence, aiding long-term planning in domains like public health and healthcare insurance.

Our results indicate that benmelstobart and anlotinib plus EC chemotherapy is not cost-effective compared with chemotherapy at the WTP threshold of $38070.59 per QALY. These findings may aid clinicians in making optimal decisions regarding the treatment of advanced ES-SCLC.

## Data Availability

The original contributions presented in the study are included in the article/Supplementary Material, further inquiries can be directed to the corresponding author.
